# Mitochondrial Dynamics in Cancer Progression and Therapy Resistance: Emerging Roles in Metabolic Reprogramming, Biomarker Discovery, and Precision Medicine

**DOI:** 10.3390/cells15111026

**Published:** 2026-06-02

**Authors:** Vasudevarao Penugurti, Rajni Kant, Che-Chia Hsu

**Affiliations:** Department of Pathology, Duke University School of Medicine, Durham, NC 27710, USA

**Keywords:** mitochondrial dynamics, mitochondrial fission, mitochondrial fusion, mitophagy, mitochondrial metabolism, oxidative phosphorylation (OXPHOS), TCA cycle, reactive oxygen species (ROS), mitochondrial DNA (mtDNA), mitochondrial dysfunction, bioenergetics, cancer, cell death, cell survival, drug resistance, immune evasion, mitochondria-targeted therapy

## Abstract

Mitochondria play essential roles in cellular metabolism and signaling, regulating biosynthetic pathways, calcium homeostasis, redox balance, and cell fate beyond ATP production. Their continual remodeling through fusion, fission, and mitophagy maintains mitochondrial quality control and adapts organelle function to cellular demands. Here, we review how mitochondrial dynamics, fusion, fission, and mitophagy modulate metabolic reprogramming and signaling to drive cancer progression and therapy resistance. Emerging evidence indicates that in cancer, mitochondrial fusion enhances respiratory efficiency and oxidative phosphorylation, whereas fission promotes glycolytic adaptation, rapid biomass accumulation, and stress tolerance. Mitophagy further refines metabolic fitness by eliminating damaged mitochondria and sustaining redox homeostasis. Together, these processes underscore that dysregulation of mitochondrial dynamics is a hallmark of cancer and a key driver of metabolic reprogramming and therapeutic resistance. In this review, we summarize how mitochondrial fusion, fission, and mitophagy govern metabolic circuitry in cancer development and therapy resistance. We highlight their functional impact on tumor progression and discuss emerging therapeutic strategies targeting mitochondrial dynamics and associated machinery. Understanding this dynamic metabolic crosstalk may reveal new vulnerabilities and guide the development of mitochondria-targeted cancer therapies.

## 1. Introduction

Mitochondria are widely recognized as the powerhouses of the cell because they generate the primary cellular energy currency, adenosine triphosphate (ATP). Beyond energy production, mitochondria regulate numerous cellular processes, including key metabolic pathways such as the tricarboxylic acid (TCA) cycle and oxidative phosphorylation (OXPHOS), as well as a few reactions in nucleotide and lipid biosynthesis, redox homeostasis, calcium signaling, apoptosis, and cell survival [1,2,3,4,5,6]. Through these interconnected metabolic networks, mitochondria integrate nutrient availability with cellular energy demands and environmental stresses such as hypoxia, nutrient deprivation, and anoikis [7,8,9,10]. In response to these challenges, cells activate adaptive metabolic programs, including glutaminolysis, fatty acid oxidation (FAO), serine–glycine metabolism, and aspartate synthesis, to sustain survival and proliferation [11,12,13,14,15,16,17,18,19]. In normal cells, mitochondrial metabolic adaptation is typically a tightly regulated and reversible process that maintains energy homeostasis in response to fluctuations in nutrient availability and substrate excess. Under physiological conditions, untransformed cells regulate metabolism through glycolysis and oxidative phosphorylation (OXPHOS) to efficiently produce ATP, while dynamically adjusting glycolytic flux, fatty acid oxidation, and mitochondrial biogenesis in response to cellular energy demands.

In contrast, malignant cells undergo persistent metabolic reprogramming characterized by enhanced aerobic glycolysis (Warburg effect) [20], altered mitochondrial respiration, and hybrid glycolytic-OXPHOS phenotypes that support rapid proliferation, survival under metabolic stress, and therapeutic resistance. These metabolic adaptations are accompanied by extensive mitochondrial remodeling, including altered fusion–fission dynamics, mitophagy, and cristae organization, which collectively enhance metabolic plasticity and stress tolerance within the tumor microenvironment.

Mitochondria continue to play central roles in cancer cell metabolism by coordinating multiple biosynthetic and bioenergetic pathways under both normal and stress conditions. For example, mitochondrial metabolism is maintained through anaplerotic reactions that replenish TCA cycle intermediates. Glutamine can be converted to glutamate and subsequently to α-ketoglutarate, thereby refilling the TCA cycle and supporting mitochondrial metabolism [21,22,23]. Similarly, cytosolic pyruvate can enter mitochondria and be converted to oxaloacetate by mitochondrial pyruvate carboxylase (PC), thereby feeding into the TCA cycle and sustaining metabolic flux [24,25].

Accumulating evidence indicates that proteins controlling mitochondrial dynamics are frequently dysregulated in cancer, where they contribute to tumor progression, metastasis, and therapy resistance. In particular, mitochondrial fission factors such as dynamin-related protein 1 (DRP1) are often overexpressed and have been implicated in cancer initiation and progression [26]. Changes in mitochondrial morphology directly affect substrate utilization, reactive oxygen species (ROS) production, mitochondrial DNA stability, mitophagy, and apoptotic sensitivity, thereby actively reshaping cellular metabolism to meet the demands of rapidly proliferating tumor cells. However, the precise mechanistic links between mitochondrial structural remodeling and metabolic rewiring remain incompletely defined and warrant further investigation.

In this review, we summarize the current understanding of how mitochondrial dynamics regulate cancer cell metabolism and metabolic reprogramming. We discuss the molecular machinery governing mitochondrial fusion and fission and examine how these processes influence cancer cell proliferation, metastasis, immune evasion, and therapeutic resistance. Finally, we highlight emerging pharmacological strategies targeting mitochondrial dynamics, with particular emphasis on their potential applications in cancer therapy. A deeper understanding of the interplay between mitochondrial structure, metabolism, and signaling may reveal novel vulnerabilities that can be exploited for therapeutic intervention in cancer.

## 2. Methodology

Relevant literature was identified through searches of PubMed, Web of Science, and Google Scholar using combinations of keywords related to mitochondrial dynamics, mitophagy, cancer metabolism, metabolic reprogramming, signaling pathways, and therapy resistance within the past 5 years. Articles published in English and relevant to the scope of this review were included. Studies were selected based on scientific quality, novelty, and relevance to the discussed molecular mechanisms and therapeutic implications. Priority was given to recent publications and to highly cited foundational studies in the field. For the expression analysis of mitochondrial dynamics proteins, we used the TIMER and GEPIA datasets and presented the corresponding histograms in the manuscript. The original data can be retrieved directly from the cited references [27,28,29,30]. Schematic figures and graphical illustrations were created using BioRender (Biorender.com) under an institutional license, in accordance with BioRender’s publication. Figures were exported at high resolution for inclusion in this review.

## 3. Overview of Mitochondrial Dynamics and Quality Control

Mitochondria undergo continuous structural remodeling through fusion, fission, mitophagy, and network reorganization, a process collectively termed mitochondrial dynamics. These dynamic actions are essential for preserving mitochondrial integrity, ensuring bioenergetic efficiency, supporting metabolic flexibility, and enabling cell survival and growth. By integrating metabolic cues, stress signals, and developmental inputs, mitochondrial dynamics shape critical cellular processes, including cell survival, apoptosis, and metabolic adaptation, thereby positioning mitochondria as central coordinators of cellular homeostasis and stress responses [31].

### 3.1. Mitochondrial Fusion

Mitochondrial fusion occurs by mixing mitochondrial contents, including mitochondrial DNA (mtDNA), proteins, lipids, and metabolites, thereby sustaining mitochondrial function and enabling adaptation to stress, such as metabolic and oxidative stress. Mechanistically, fusion occurs in two coordinated steps that involve the merging of the outer and inner mitochondrial membranes. Outer membrane fusion is mediated by the large GTPases Mitofusin 1 (MFN1) and Mitofusin 2 (MFN2), which form homotypic or heterotypic dimers of MFN1 and MFN2 between two different mitochondria to tether and merge their outer membranes [32].

**Figure 1:**
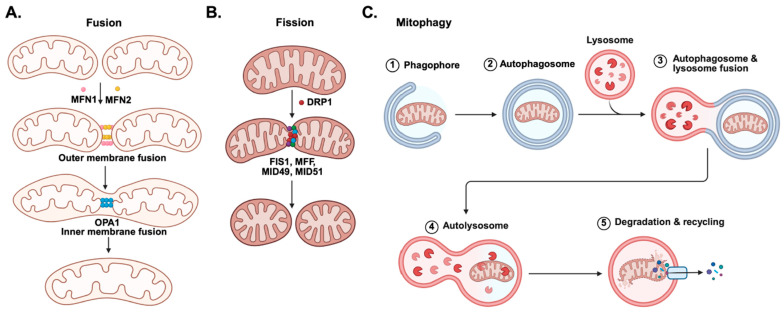
Mitochondrial dynamics and quality control: fission, fusion, and mitophagy. (**A**) Mitochondrial fusion occurs through the coordinated action of mitofusins (MFN1 and MFN2), which regulate outer mitochondrial membrane fusion, whereas OPA1 mediates inner mitochondrial membrane fusion. (**B**) Mitochondrial fission is primarily regulated by DRP1 (DNM1L). Adaptor proteins, including MFF, FIS1, MID49, and MID51, recruit and facilitate DRP1 assembly on the mitochondrial surface, leading to mitochondrial fragmentation. (**C**) Mitophagy is a mitochondrial quality control mechanism involving the selective degradation of damaged mitochondria through autophagy. Damaged mitochondria are sequestered into autophagosomes, which subsequently fuse with lysosomes to form autolysosomes, where mitochondrial components are degraded. Figures were created using BioRender (BioRender.com).

Next, inner membrane fusion is regulated by Optic Atrophy 1 (OPA1), a dynamin-related GTPase localized to the inner mitochondrial membrane (Figure 1A) [33]. OPA1 not only mediates fusion of the inner membrane but also governs cristae architecture, thereby influencing the organization of the respiratory chain and controlling cytochrome c sequestration within the cristae during physiological conditions. Under pro-apoptotic stimuli, OPA1-dependent cristae remodeling modulates the accessibility of cytochrome c and facilitates its release, thereby shaping the amplitude and timing of apoptosis.

Mitochondrial fusion is functionally associated with increased OXPHOS, enhanced mitochondrial membrane potential, efficient ATP production, and resistance to cellular stress [34,35]. Fused mitochondria often support anabolic metabolism and are observed in cells with great energy demand [34].

### 3.2. Mitochondrial Fission

In contrast, mitochondrial fission generates smaller, discrete mitochondria and is essential for mitochondrial distribution during cell division, quality control, and mitophagy. Increased mitochondrial fragmentation is frequently associated with elevated ROS production, increased glycolytic metabolism, activation of mitophagy, and apoptotic priming [36].

Mitochondrial fission occurs through the binding of activated DRP1, a cytosolic GTPase, to the adaptor proteins such as Fission1 (FIS1), Mitochondrial Fission Factor (MFF), Mitochondrial dynamics protein 49 (MID49), and Mitochondrial dynamics protein 51 (MID51) on the outer mitochondrial membrane. After DRP1 binds to these adaptor proteins, it oligomerizes and constricts the membrane to drive scission (Figure 1B) [37,38,39,40]. It has been reported that DRP1 undergoes various post-translational modifications, including phosphorylation by different kinases, SUMOylation, ubiquitination, and acetylation, which fine-tune its activity in response to metabolic and stress signals [41,42,43,44,45].

### 3.3. Mitophagy and Mitochondrial Biogenesis

In addition to fusion and fission, mitophagy and mitochondrial biogenesis also contribute to mitochondrial homeostasis by regulating mitochondrial biomass, respiratory capacity, and metabolic function. These processes are closely associated with mitochondrial dynamics because, like fusion and fission, they contribute to maintaining mitochondrial quality control, remodeling the mitochondrial network, and preserving cellular energy balance. Coordinated regulation among mitochondrial dynamics, mitophagy, and mitochondrial biogenesis is therefore essential for sustaining mitochondrial integrity and cellular metabolic function.

To preserve mitochondrial quality and functionality, cells employ mitophagy, a specialized form of selective autophagy that targets damaged or superfluous mitochondria for degradation. In this process, defective organelles are selectively recognized and sequestered by the phagophore, an isolation membrane that progressively engulfs the mitochondrion to form a double-membrane autophagosome. The autophagosome subsequently fuses with lysosomes, forming an autophagolysosome, where lysosomal hydrolases degrade mitochondrial proteins, lipids, and nucleic acids [46,47]. The resulting metabolites are recycled back into the cytosol to support cellular metabolism, thereby reinforcing mitochondrial quality control and contributing to metabolic adaptation (Figure 1C).

Mitochondrial biogenesis is tightly integrated with mitochondrial dynamics and mitophagy to maintain mitochondrial homeostasis. Biogenesis, driven largely by PGC-1α/PGC-1β and downstream transcription factors such as NRF1, NRF2, and TFAM, increases mitochondrial mass and promotes the synthesis and import of nuclear-encoded mitochondrial proteins in response to energetic stress, nutrient cues, and oncogenic signaling. This increase in mitochondrial content must be balanced by fusion and fission events, which distribute newly formed organelles throughout the network, allow mixing of mitochondrial DNA and proteins, and segregate damaged segments for mitophagy. In cancer cells, upregulated biogenesis can cooperate with fusion to generate highly interconnected, OXPHOS-competent networks that support anabolic growth and therapy resistance, whereas imbalanced biogenesis and fission may lead to fragmented, ROS-prone networks that favor glycolytic adaptation and stress signaling. Thus, mitochondrial biogenesis not only adjusts mitochondrial quantity but also indirectly shapes mitochondrial morphology, distribution, and quality, reinforcing the view that biogenesis, dynamics, and mitophagy form an integrated circuit controlling mitochondrial function in cancer [48].

### 3.4. Regulation by Cellular Signals

Mitochondrial dynamics are regulated by nutrient availability, hypoxia, oncogenic cues, calcium flux, and energy-sensing pathways such as AMP-activated protein kinase (AMPK) and mechanistic target of rapamycin (mTOR) [17,49,50,51,52]. Beyond structural remodeling, these signals are transduced through changes in mitochondrial fusion and fission to reprogram cellular metabolism, survival, and stress adaptation. Energy-sensing pathways converge on mitochondrial dynamics, with AMPK activation often promoting fission and mitophagy under low-energy conditions, while mTOR favors fusion and anabolic growth [49,53,54,55]. AMP-activated kinase (AMPK) is a stress response kinase activated under metabolic stress and promotes mitochondrial fission. A study showed that AMPK phosphorylates Inverted formin 2 (INF2) at Ser1077, leading to increased localization of INF2 and DRP1 to the ER and promoting fission [53]. In another study, mitofusion mediated by MFN2 promotes AMPK activation and cancer progression [55]. Other factors, such as Transcriptional coactivators like Peroxisome proliferator-activated receptor gamma coactivator-1 α/β (PGC1α/PGC1β), control the expression of MFN1, MFN2, and other mitochondrial genes, thereby linking growth and oncogenic signaling, such as Yes-associated protein (YAP)/Hippo-related pathways, to mitochondrial fusion and oxidative metabolism [56]. Phosphatase and Tensin Homolog (PTEN)-induced kinase 1 (PINK1) and Parkin (PRKN) orchestrate mitophagy, which in turn shapes ROS signaling, inflammasome activation, and apoptosis, and is itself regulated by oncogenic and stress-response pathways [57]. FIS1, MFF, and Mitochondrial Rho (MIRO) family members act as receptors that couple fission and mitophagy to calcium and kinase signaling, integrating organelle remodeling with cell survival decisions [58]. Not only cellular signals, proteins, and oncogenes regulate mitochondrial dynamics, but metabolites do as well. Inositol also suppresses mitochondrial fission by inhibiting AMPK-mediated DRP1 phosphorylation [49]. Lactate and Fumarate are also shown to regulate mitochondrial dynamics [59,60].

Taken together, mitochondrial fusion-regulated genes, fission-regulated genes, PGC1α/β, AMPK, and mTOR form a gene network by which mitochondrial dynamics transduce nutrients, metabolite-derived signals, stress, and oncogenic signals into metabolic rewiring, ROS signaling, and cell-fate outcomes. Thus, mitochondrial dynamics act as a central signaling hub, linking extracellular and intracellular stress cues to metabolic control, cell-fate decisions, and survival pathways.

## 4. Mitochondrial Dynamics Regulate the Cancer Cell Metabolism

Mitochondrial dynamics are increasingly recognized not only as structural remodeling processes but also as active regulators of metabolic reprogramming in cancer. The balance between fission and fusion defines mitochondrial architecture, cristae organization, substrate utilization, and respiratory efficiency. Through these mechanisms, mitochondrial dynamics directly influence metabolic pathway selection, biosynthetic capacity, redox balance, and tumor cell adaptability. In this section, we will discuss how mitochondrial dynamics modulate metabolic reprogramming and cellular signaling transduction, and highlight mitochondrial dynamics in various metabolic pathways required for cancer cell survival and proliferation.

### 4.1. OXPHOS and Glycolysis

**Figure 2:**
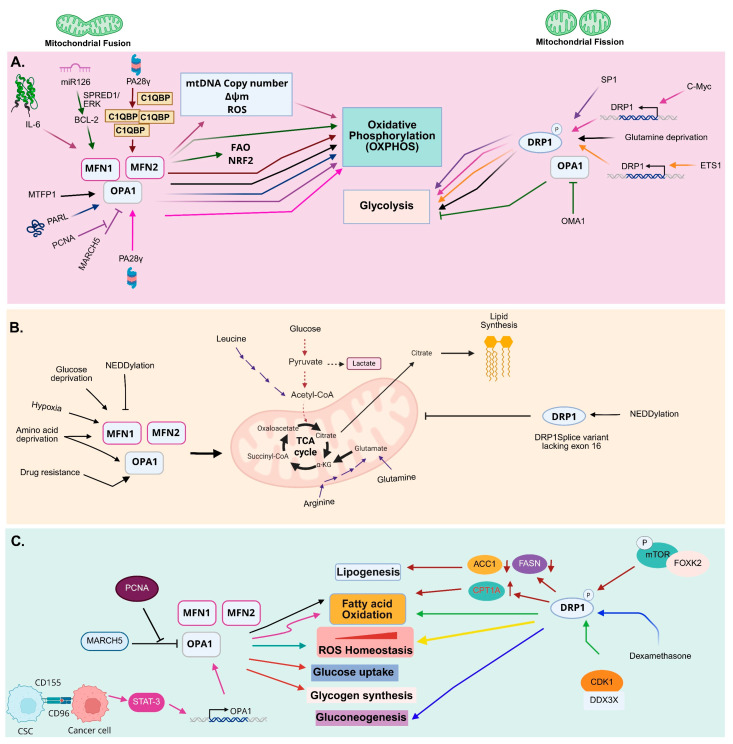
Role of mitochondrial dynamics in cancer cell metabolism. (**A**) Mitochondrial dynamics, including fusion and fission, play a central role in regulating cellular metabolism. Mitochondrial fusion is generally associated with enhanced oxidative phosphorylation (OXPHOS), whereas fission is often linked to increased glycolytic activity. (**B**) Mitochondrial dynamics influence the tricarboxylic acid (TCA) cycle and anaplerosis by replenishing metabolic intermediates, particularly under conditions of energy, metabolic stress, or drug resistance. (**C**) Mitochondrial dynamics regulate multiple metabolic pathways, including fatty acid metabolism, reactive oxygen species (ROS) homeostasis, glycogen synthesis, glucose uptake, and gluconeogenesis. Each colored arrow/inhibitory line indicates the specific pathway that it regulates. Figures were created using BioRender (BioRender.com).

Elongated mitochondria are associated with improved cristae organization, enhanced assembly of electron transport chain (ETC) supercomplexes, increased mitochondrial membrane potential (Δψm), and elevated mitochondrial DNA (mtDNA) copy number, collectively promoting efficient ATP production and enhanced oxidative phosphorylation (OXPHOS). Mitochondrial fusion proteins, including MFN1, MFN2, and OPA1, play critical roles in regulating these processes across various cancer models. In several cancers, the formation of fused mitochondrial networks supports high OXPHOS activity, thereby facilitating metastatic dissemination, therapy resistance, and the maintenance of cancer stem cell properties. Pro-inflammatory cytokines such as interleukin-6 (IL-6) play a major role in cancer progression by regulating mitochondrial dynamics. IL-6 upregulates MFN1 expression, which, in turn, induces a depolarization of the mitochondrial membrane potential (Δψm) and an increase in mtDNA copy number, thereby facilitating OXPHOS in acute myeloid leukemia (AML) [61]. MicroRNAs play a major role in regulating mitochondrial dynamics. MicroRNA-126 (miR-126) promotes OXPHOS by stabilizing B-cell lymphoma 2 (BCL-2) through the Sprouty-related, EVH1 domain-containing protein 1 (SPRED1)/extracellular signal-regulated kinase axis, which in turn promotes phosphorylation of MFN1/2 to induce mitofusion and suppress mitofission by regulating DRP1 phosphorylation [62]. Proteasomal components also regulate mitochondrial dynamics. Proteasome activator 28γ (PA28γ) is a member of the 20S proteosome complex, and an ATP- and ubiquitin-independent proteasome regulator that regulates the OXPHOS by binding and stabilizing the complement 1q binding protein (C1QBP), which regulates the MFN1/2 and OPA1 expression [63]. OPA1 is another piece of machinery in mitofusion, and its role in maintaining cristae integrity is particularly critical for respiratory capacity. The studies suggest that OPA1 is overexpressed in AML, suppresses ROS production, enhances metabolic reprogramming by increasing OXPHOS, and suppresses apoptosis [64]. E3 ligases play an important role in regulating mitochondrial dynamics. Membrane Associated Ring-CH-Type Finger 5 (MARCH5) is the mitochondrial outer membrane E3 ubiquitin–protein ligase that is essential for maintaining mitochondrial homeostasis, which degrades OPA1 in cancer cells. Proliferating cell nuclear antigen (PCNA) is highly overexpressed in AML and binds to OPA1, inhibiting MARCH5 binding to OPA1 and promoting mitofusion and OXPHOS [65]. Certain proteases were known to be involved in and regulate mitochondrial dynamics. Presenilin-associated rhomboid-like protein (PARL), which is an inner-mitochondrial-membrane rhomboid protease, maintains mitochondrial homeostasis by cleaving specific proteins in apoptosis, supports fusion-competent and elongated mitochondria via OPA1 processing, thereby supporting efficient electron transport and OXPHOS [66] (Figure 2A).

In contrast, mitochondrial fission is often associated with a metabolic shift toward glycolysis. DRP1-mediated fragmentation reduces respiratory efficiency and promotes metabolic rewiring toward aerobic glycolysis, facilitating rapid ATP production and biomass synthesis needed for proliferation. Additionally, mitochondrial fission can be triggered under nutrient deprivation. Glutamine is an essential component for proliferating cells, required for DNA synthesis and anaplerotic reactions. Glutamine deprivation conditions induce ROS-mediated activation of the Mitogen-activated protein kinase (MAPK)-extracellular signal-regulated kinase 1/2 (ERK1/2) signaling pathway, leading to further phosphorylation of DRP1 at Serine 616, increased glycolysis, unaltered OXPHOS, and the promotion of cancer stem cell formation [67].

Fragmented mitochondria are commonly observed in highly proliferative tumors and under oncogenic signaling [68,69]. At the molecular level, DRP1 binding is increased by FIS1 rather than MFF, promoting mitophagy, mitochondrial respiratory cristae (MRC) dynamics, OXPHOS, and ATP production [69]. C-Myc is another important oncogene that plays a key role in rewiring cancer cell metabolism by regulating glycolysis, glutamine metabolism, and mitochondrial biogenesis, while also fine-tuning lipid and nucleotide synthesis [19]. In a study using hepatoblastoma models, it was shown that C-Myc promotes glycolysis by upregulating DRP1, which increases mitofission; this enhanced mitofission increases the glycolytic phenotype in cells and suppresses MFN1 expression by increasing miR-373-3p, which leads to reduced mitofusion [68]. Some aggressive tumors, such as gliomas and ovarian tumors, exhibit high fission alongside preserved mitochondrial metabolism, underscoring that mitochondrial fragmentation can enhance metabolic plasticity rather than suppress respiration [69,70]. Components of the electron transport chain (ETC) also regulate mitochondrial dynamics by modulating fission and fusion proteins. A study showed that ETS Proto-Oncogene 1 (ETS1), a transcription factor, directly binds to the DRP1 promoter, increasing its expression and promoting mitofission and enhanced glycolysis rather than OXPHOS [70]. Another component of mitochondria is Overlapping Activity With M-AAA Protease (OMA1), a stress-activated protease that regulates the balance between cell survival and death during metabolic stress. It cleaves long-form OPA1 into short isoforms, inhibiting inner-membrane fusion and promoting mitochondrial fragmentation. This stress-induced fragmentation and reduced fusion correlate with and can promote a metabolic shift toward glycolysis, helping cells cope with energetic or oxidative stress [71]. This glycolytic bias supports nucleotide, amino acid, and lipid biosynthesis by diverting glycolytic intermediates into anabolic pathways. Thus, mitochondrial morphology acts as a rheostat that balances OXPHOS-dependent energy production with glycolysis-driven biomass production (Figure 2A).

In summary, mitochondrial fusion improves respiratory efficiency and OXPHOS, while fission supports glycolytic adaptation, rapid biomass accumulation, and stress tolerance. Nevertheless, this relationship is context-dependent. Mitofusion not only regulates the OXPHOS but also regulates glucose metabolism. Recent studies suggest that the specificity protein 1 (Sp1) regulates mitochondrial dynamics through MFN1/2, OPA1, and DRP1, and promotes glycolysis [72]. Mitochondrial Fission Process 1 (MTFP1), which participates in the classical fission machinery, serves as a regulator of inner-membrane integrity to promote a glutamine-driven OXPHOS axis [73]. Therefore, this fusion–fission-driven metabolic reprogramming appears to be context-dependent and may allow cancer cells to adapt to different stimuli.

### 4.2. TCA Cycle and Anaplerosis

Beyond OXPHOS and glycolysis, mitochondrial dynamics also influence TCA cycle flux and anaplerotic pathways that replenish metabolic intermediates. Metabolites produced by the TCA cycle have been linked to cancer progression, and this progression depends on mitochondrial dynamics. Fused mitochondria tend to support robust TCA cycling, sustaining citrate production for export into the cytosol. Cytosolic citrate serves as a precursor for lipid synthesis and histone acetylation, linking mitochondrial dynamics to epigenetic and biosynthetic programs [74]. During metabolic stress, cancer cells utilize alternative metabolic pathways, a process known as metabolic reprogramming. Mitochondrial dynamics also regulate metabolic reprogramming, including glutamine import and metabolism, a major anaplerotic input in cancer cells. Glutamine-derived α-ketoglutarate fuels the TCA cycle and supports nucleotide and lipid biosynthesis [75]. In another type of metabolic stress, such as amino acid starvation, cells increase hyperfusion via MFN1 and OPA1. Supplementation with glutamine, leucine, and arginine further increases hyperfusion, and knockdown of genes regulating glutaminolysis, the TCA cycle, and purine biosynthesis indicates that hyperfusion is involved in these pathways [76]. Hypoxia, a condition in which cells or tissues experience low oxygen levels compared to normal physiological conditions, where cancer cells frequently utilize reductive carboxylation of glutamine-derived α-ketoglutarate to generate citrate for lipid production [77]. Another metabolic adaptation, where fragmented mitochondria may favor cell growth by reshaping the nicotinamide adenine dinucleotide (NAD+/NADH) balance and reorganizing metabolic enzyme localization [78]. Not only these conditions, but also certain cellular signaling pathways regulate TCA cycle activity through modulation of mitochondrial dynamics. For example, post-translational modifications such as NEDDylation, in which Neural precursor cell expressed developmentally downregulated protein 8 (NEDD8) is conjugated to target proteins, regulate protein stability and mitochondrial function. Inhibition of NEDDylation stabilizes MFN1 and prevents the translocation of the fission protein DRP1 to mitochondria, thereby promoting mitochondrial fusion while suppressing TCA cycle activity [79]. Similarly, alterations in mitochondrial dynamics proteins and their regulatory mechanisms further influence TCA cycle activity and metabolic reprogramming in cancer cells. Drug-resistant and residual cancer cells often increase the TCA cycle and reprogram metabolic pathways through OPA1-mediated hyperfusion [80]. Further, the DRP1 splice variants were shown to regulate mitofusion. DRP1 splice variant lacking exon 16, has defects in the fission events and promotes the fusion events, enhances the TCA cycle metabolites, and increases the metastasis [81,82,83]. Through these mechanisms, mitochondrial dynamics regulate the flexibility of TCA cycle engagement and biosynthetic output (Figure 2B).

### 4.3. Fatty Acid Metabolism

Fatty acid metabolism plays an essential role in cancer cell survival under metabolic stress conditions. Fatty acid oxidation (FAO), which occurs in the mitochondria, generates energy to support cellular survival, whereas fatty acid synthesis is often suppressed during stress to conserve energy. Mitochondrial dynamics regulate these metabolic processes by modulating mitochondrial function, bioenergetic flexibility, and stress adaptation, thereby promoting cancer cell survival under adverse conditions. Fused mitochondrial networks are often associated with enhanced FAO, as elongated mitochondria exhibit improved respiratory coupling and sustained oxidative capacity [84]. FAO provides ATP and NADH/flavin adenine dinucleotide (FADH2) to support tumor survival under nutrient limitation, anchorage-independent growth, and therapeutic resistance [84]. Cluster of Differentiation 96 (CD96) is highly overexpressed in various cancers and enhances mitochondrial FAO via the CD155-CD96-Src-Signal Transducer and Activator of Transcription 3 (Stat3)-Opa1 pathway in breast cancer models [85]. OPA1 has also been shown to increase fatty acid oxidation (FAO) in Acute Myeloid Leukemia. PCNA binds to OPA1 and prevents its interaction with the E3 ubiquitin ligase MARCH5, thereby protecting OPA1 from MARCH5-mediated degradation [65]. In addition, mitochondrial dynamics regulate interactions between mitochondria and lipid droplets. Physical contact sites between these organelles facilitate fatty acid trafficking for either β-oxidation or lipid storage [86]. In metastatic breast cancer cells, elevated lipid content promotes FAO. This metabolic adaptation is regulated by DRP1-mediated mitochondrial fission. Phosphorylation of DRP1 by cyclin-dependent kinase 1 (CDK1) stimulates mitochondrial fission, a process that is coordinated by the DEAD-box helicase 3 (DDX3) [86]. In another study, DRP1 was shown to regulate fatty acid oxidation (FAO) by interacting with the mTOR signaling pathway. Forkhead box protein K2 (FOXK2) increases mTOR phosphorylation and interacts with both mTOR and DRP1, thereby promoting lipid metabolic reprogramming. FOXK2-mediated activation of the mTOR-DRP1 axis enhances the expression of carnitine palmitoyltransferase 1A (CPT1A), a key enzyme involved in mitochondrial fatty acid transport and FAO, while reducing the expression of lipogenic enzymes such as fatty acid synthase (FASN) and acetyl-CoA carboxylase 1 (ACC1) [87]. In certain cancers, fragmented mitochondria localize near lipid droplets, promoting rapid lipid mobilization to meet biosynthetic demands. Conversely, fusion can enhance sustained FAO-dependent survival programs. Taken together, mitochondrial dynamics govern not only OXPHOS but also lipid utilization strategies by increasing mitochondrial dynamics and substrate accessibility, thereby contributing to tumor aggressiveness and metabolic resilience (Figure 2C).

### 4.4. Redox Homeostasis and ROS Signaling

Mitochondrial dynamics play an important role in maintaining cellular redox balance and regulating ROS signaling. Changes in mitochondrial morphology not only alter total ROS levels but also shape localized ROS microdomains by reorganizing cristae structure, respiratory chain supercomplexes, and local oxygen consumption within the mitochondrial network. In fragmented mitochondria, increased curvature, disrupted cristae, and uneven distribution of ETC components can reduce electron transfer efficiency, leading to focal sites of electron leakage at complexes I and III and the formation of ROS “hotspots” along discrete mitochondrial fragments [88]. These localized ROS microdomains can converge on nearby redox-sensitive targets, such as ion channels, kinases, and phosphatases, at mitochondria-ER or mitochondria-plasma membrane contact sites to modulate calcium flux, membrane potential, and downstream signaling with high spatial specificity.

Mitochondrial fission is often associated with elevated ROS levels because shortened, depolarized segments show impaired NADH oxidation and increased electron leakage from the ETC. Although excessive ROS can damage lipids, proteins, and DNA and trigger cell death, cancer cells frequently upregulate antioxidant systems (for example, glutathione, thioredoxin, and NRF2-driven programs) and survival pathways to buffer these ROS microdomains, allowing ROS to function as compartmentalized signaling cues rather than purely toxic species. In contrast, mitochondrial fusion generally supports a more homogeneous cristae organization and more efficient electron transport, thereby distributing electron flux and ROS production across an interconnected network. This architecture facilitates metabolic “sharing” and functional complementation between mitochondria, enhances redox buffering capacity, and limits the formation of highly concentrated ROS foci, thereby stabilizing mitochondrial and cellular redox homeostasis.

Physiologically, low to moderate ROS generated in specific mitochondrial subdomains act as second messengers that regulate redox-sensitive metabolic regulators and transcription factors, including AMP-activated protein kinase (AMPK), Nuclear factor erythroid 2-related factor 2 (NRF2), MYC, and p53, thereby linking mitochondrial structure to broader metabolic control and stress adaptation [89,90,91]. Local ROS surges in hypoxic or nutrient-limited tumor regions can also stabilize hypoxia-inducible factors (HIFs) and activate pro-angiogenic and pro-metastatic transcriptional programs, further illustrating how architecture-dependent ROS microdomains shape cancer cell behavior. Importantly, oxidative stress can directly activate DRP1, enhancing mitochondrial fission and promoting additional fragmentation. This establishes a positive feedback loop in which mitochondrial fragmentation amplifies localized ROS production, which, in turn, further drives fission and redox-sensitive oncogenic signaling [75,88]. Conversely, a shift toward mitochondrial fusion can dampen these ROS microdomains by improving ETC coupling, promoting cristae integrity, and increasing the network’s capacity to dissipate oxidative stress.

Thus, the balance between mitochondrial fission and fusion not only sets the overall level of mitochondrial ROS but also determines the spatial patterning and signaling specificity of ROS microdomains. This architectural control is critical in cancer cells, where finely tuned ROS signals can promote metabolic reprogramming, proliferation, and invasion, whereas excessive or poorly controlled ROS can overwhelm antioxidant defenses and lead to cell death (Figure 2C) [69].

### 4.5. Glycogen Metabolism, Gluconeogenesis, and De Novo Purine Synthesis

It has been reported that mitochondrial dynamics regulate additional metabolic pathways beyond classical energy metabolism, including glycogen metabolism, gluconeogenesis, and de novo purine synthesis. In cells with DRP1 knockdown, enhanced mitochondrial fusion is observed [62,75]. For example, Drp1 loss in colon cancer cells reduces mitochondrial respiration and diverts glucose toward glycogen synthesis via AMPK-activation of glycogen synthase (GYS1), leading to glycogen accumulation and enhanced cell survival [92]. These findings suggest that alterations in mitochondrial morphology can regulate glucose utilization and glycogen storage pathways. Glucocorticoids such as dexamethasone have been shown to regulate key gluconeogenic enzymes, including pyruvate carboxylase, phosphoenolpyruvate carboxykinase (PEPCK), and glucose-6-phosphatase. In addition, glucocorticoids promote the breakdown of fats and proteins, thereby releasing glycerol and amino acids that the liver can utilize as substrates for gluconeogenesis [93,94]. In a study, dexamethasone induced DRP1 expression, leading to mitochondrial fission and promoting gluconeogenesis through metabolic adaptation toward glucose production [95]. In the context of amino acid availability, amino acid deprivation triggers mitochondrial hyperfusion mediated by MFN1 and OPA1, which helps maintain mitochondrial ATP production and cellular survival under nutrient stress, whereas supplementation with glutamine, leucine, or arginine further enhances this hyperfused state [76]. Overall, these studies demonstrate that mitochondrial dynamics directly influence multiple metabolic pathways by modulating mitochondrial structure, bioenergetics, nutrient utilization, and biosynthetic adaptation in cancer cells (Figure 2C).

### 4.6. Direct Signaling Metabolites Pathway

Recent studies further demonstrate that metabolic intermediates can directly regulate mitochondrial dynamics. For example, in lenvatinib-resistant HCC, elevated lactate production, driven by enhanced glycolytic metabolism, was shown to induce lactylation-dependent mitochondrial translocation of α/β hydrolase domain-containing 6 (ABHD6). Mitochondrial-localized ABHD6 functioned as a scaffold protein that disrupted DRP1-FIS1-mediated mitochondrial fission, thereby reducing ROS production [96]. In another study, miR-373-3p, a microRNA targeting MFN2, induces glycolysis and lactate production, thereby promoting cancer cell proliferation [97]. These findings highlight how lactate functions not only as a metabolic byproduct but also as a signaling mediator linking metabolic reprogramming and mitochondrial dynamics in cancer.

### 4.7. Metabolic Cooperation Under the Tissue-Specific Microenvironment

Mitochondrial dynamics are not regulated solely by intrinsic oncogenic signaling pathways but are also strongly influenced by tissue-specific tumor microenvironments and metabolic interactions with surrounding stromal and somatic cells. Increasing evidence indicates that metabolic cooperation between cancer cells and surrounding stromal or somatic cells, particularly cancer-associated fibroblasts (CAFs), directly fuels tumor growth and shapes mitochondrial function. CAFs and related stromal populations secrete metabolites, such as lactate, pyruvate, alanine, glutamine, and fatty acids, which are imported by cancer cells and used as alternative carbon and nitrogen sources to sustain the TCA cycle, OXPHOS, and lipid biosynthesis under stress conditions [98]. These stromal-derived nutrients, together with extracellular matrix remodeling and autophagy in the tumor stroma, have been shown to modulate mitochondrial morphology, increase mitochondrial biogenesis and respiratory capacity, and fine-tune redox balance in a context-dependent manner, thereby promoting metabolic flexibility, invasiveness, and therapy resistance [99].

## 5. Mitochondrial Dynamics and Cancer Cell Phenotypes

Mitochondrial dynamics influence several cancer cell phenotypes. Fission driven by increased DRP1 activity fragments mitochondria, reducing OXPHOS while enhancing glycolysis (Warburg effect), which may promote cancer cell proliferation and invasion [100]. Fusion, mediated by elevated MFN1/2 or OPA1 levels, produces elongated mitochondria that increase OXPHOS, driving differentiation and cell survival [100]. However, mitochondrial dynamics can regulate cancer phenotypes through mechanisms beyond metabolic reprogramming, including effects on cell cycle progression, apoptosis resistance, ROS signaling, mitophagy, and DNA damage responses.

**Figure 3:**
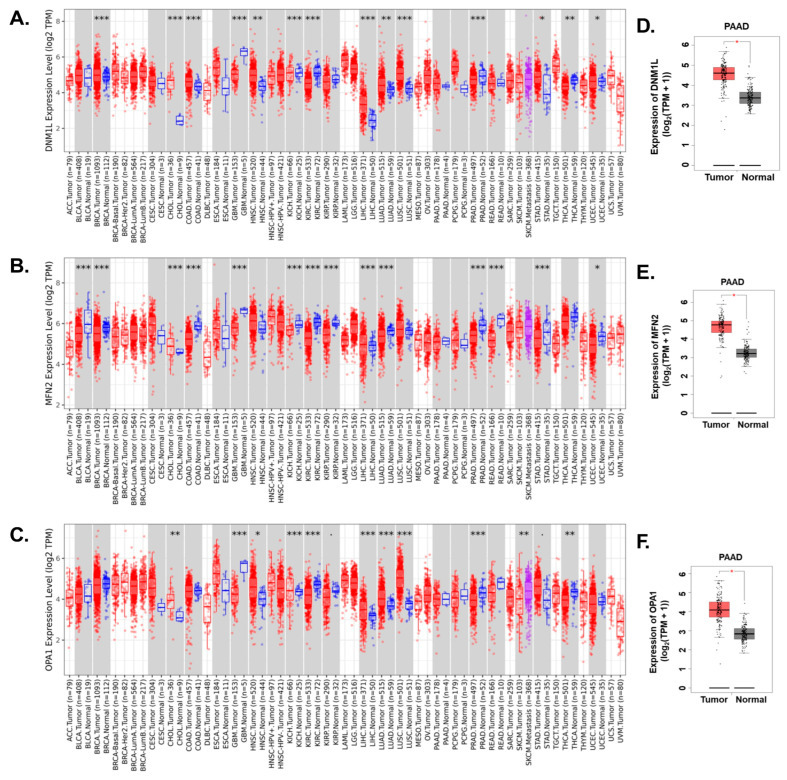
Expression profile of mitochondrial dynamics machinery across cancers. (**A**) Expression profile of DRP1 (DNM1L) across multiple cancer types (data retrieved from TIMER datasets). (**B**) Expression profile of MFN2 across multiple cancer types (TIMER datasets). (**C**) Expression profile of OPA1 across multiple cancer types (TIMER datasets). (**D**–**F**). Expression levels of DRP1, MFN2, and OPA1 in normal and pancreatic adenocarcinoma (PAAD) (Tumor) samples, a highly aggressive malignancy characterized by extensive metabolic reprogramming and dysregulation of mitochondrial dynamics. Asterisks indicate statistical significance: *p* < 0.05 (*), *p* < 0.01 (**), *p* < 0.001 (***). Different colors and grey shading are used to distinguish cancer and normal samples, as well as different subtypes within each cancer group, as indicated in the figure.

A conserved set of dynamin-related GTPases, including DRP1, MFN1, MFN2, and OPA1, governs mitochondrial morphology. These proteins were initially characterized as structural regulators of mitochondrial networks but are now recognized as key modulators of intracellular signaling and apoptotic sensitivity. For example, increased DRP1-dependent fission has been linked to proliferation and tumor formation, while DRP1 inhibition or MFN1/2 overexpression can reduce growth and trigger apoptosis in cancer cells [16,101,102]. We also analyzed increased expression of these mitochondrial dynamics proteins, such as DRP1 (DNM1L), MFN2, and OPA1, using the GEPIA [27] and TIMER [28,29,30] databases across various cancers compared with normal patient samples (Figure 3), indicating that fission and fusion-regulated proteins serve as regulators of signaling pathways and affect tumor growth and stress adaptation directly.

A growing body of evidence shows that mitochondrial dynamics influence not only metabolism but also core cancer cell phenotypes, including proliferation, stemness, invasion, metastasis, and resistance to cell death. Changes in mitochondrial morphology can alter ROS production, apoptotic sensitivity, mitophagy, and signaling pathway activation, thereby reshaping tumor behavior in both early and advanced disease. In many cancers, fusion and fission are co-opted as adaptive programs that support growth under stress and promote malignant progression. In this section, we discuss how mitochondrial dynamics regulate these phenotypic traits through metabolic rewiring and signaling crosstalk.

### 5.1. Proliferation and Tumor Growth

Metabolic reprogramming can also regulate tumor growth and cell proliferation through mitochondrial dynamics. Mitochondrial outer and inner membrane proteins are involved in cell proliferation and tumor growth. FUN14 domain-containing protein 2 (FUNDC2) is a mitochondrial outer membrane protein associated with mitochondrial quality control, stress signaling, and mitophagy-related pathways. FUNDC2 is highly overexpressed in various cancers and promotes mitochondrial fragmentation, leading to metabolic reprogramming [103]. Transcription factors regulate cell proliferation and tumor progression by modulating mitochondrial dynamics. Specificity Protein (SP1) is highly upregulated in various cancers and contributes to mitochondrial homeostasis by transcriptionally regulating genes involved in mitochondrial biogenesis, oxidative metabolism, and stress-responsive signaling pathways. SP1 also influences mitochondrial remodeling by modulating the expression of MFN1/2, OPA1, and DRP1, thereby altering the balance between mitochondrial fusion and fission. These changes promote aerobic glycolysis, enhance cancer cell proliferation, and suppress apoptosis [72]. Proteasome components also regulate mitochondrial dynamics and promote cell growth and proliferation. For example, Proteasome activator 28γ (PA28γ) is a nuclear proteasome activator that mediates ubiquitin-independent protein degradation by activating the 20S proteasome. PA28γ has been identified as a regulator of mitochondrial dynamics by colocalizing with complement 1q binding protein (C1QBP) in mitochondria, thereby promoting mitochondrial fusion, enhancing oxidative phosphorylation (OXPHOS), and contributing to the progression of oral squamous cell carcinoma (OSCC) [63]. OPA1, a mitochondrial inner membrane protein, plays a critical role in cell proliferation by regulating mitochondrial fusion and respiration [104]. In another study, OPA1 was shown to be regulated by T-cell immunoglobulin and mucin domain-containing molecule 4 (TIM-4), which promotes OXPHOS. Functionally, TIM-4 interacts with annexin A2 (ANXA2) and activates the phosphatidylinositol 3-kinase (PI3K)/Protein kinase B (AKT) pathway, thereby increasing tumor growth [105].

Mitochondrial dynamics support cancer cell proliferation beyond metabolic reprogramming. For instance, elevated activity of DRP1 has been reported in several malignancies, including hepatocellular carcinoma (HCC) [106], breast cancer [107], and glioblastoma [108], where it correlates with enhanced tumor growth and poor prognosis. Activation of DRP1 is tightly regulated by post-translational modifications, particularly phosphorylation by cell cycle kinases such as Cyclin-dependent kinase 1 (CDK1) and cyclin B during the G2/M transition [109]. Proteasome non-ATPase regulatory subunit 14 (PSMD14), a deubiquitinating enzyme, regulates DRP1 activity. Increased levels of PSMD14 have been observed in bladder cancer, where it promotes cell growth through DRP1-mediated mitochondrial fission [69]. Another regulator, the E3 ubiquitin ligase Mulan1 (MUL1), suppresses cervical cancer growth. Specifically, MUL1 promotes the ubiquitination of FUN14 domain-containing 1 (FUNDC1), an activator of DRP1 [110]. Exosomes act as carriers of oncogenic proteins and mitochondrial regulatory factors, thereby modulating mitochondrial dynamics and promoting cancer cell growth and proliferation. For example, exosomal 4EBP1 derived from the serum of head and neck cancer (HNC) patients promotes cell proliferation and migration. From a mechanistic perspective, exosomal 4EBP1 enhances cancer progression by regulating DRP1 and FIS1 [111]. Mitochondrial dynamics proteins can also influence transcriptional programs that promote cancer progression. In some contexts, DRP1 functions in transcriptional regulation by modulating the activity of Forkhead box protein M1 (FOXM1), a transcription factor that regulates Matrix metalloproteinase-12 (MMP12) expression, thereby contributing to proliferation and metastasis in head and neck cancer (HNC) [112]. Mitochondrial electron transport chain (ETC) components also play important roles in regulating mitochondrial dynamics and cancer progression. For example, NADH: ubiquinone oxidoreductase subunit AB1 (NDUFAB1) is highly overexpressed in HCC and promotes mitochondrial fusion by upregulating MFN1/2 and OPA1 while downregulating DRP1. This shift activates mitophagy and supports cancer progression [113].

Mitophagy serves as an important mitochondrial quality control mechanism that enables cancer cells to survive under hypoxic and metabolic stress conditions by removing damaged mitochondria and maintaining cellular bioenergetic homeostasis. A recent study demonstrated that hypoxia induces Hypoxia-inducible factor 1-alpha (HIF1α)-mediated upregulation of Glycerophosphocholine Phosphodiesterase 1 (GPCPD1), which is subsequently depalmitoylated by Lysophospholipase 1 (LYPLA1). Depalmitoylated GPCPD1 translocates to mitochondria, where it interacts with Voltage-Dependent Anion Channel 1 (VDAC1) and promotes PRKN-mediated mitophagy. This process facilitates the selective removal of dysfunctional mitochondria, thereby reducing mitochondrial stress and reactive oxygen species (ROS) accumulation, maintaining mitochondrial function, and enhancing cancer cell adaptation and survival under hypoxic conditions [114].

**Figure 4:**
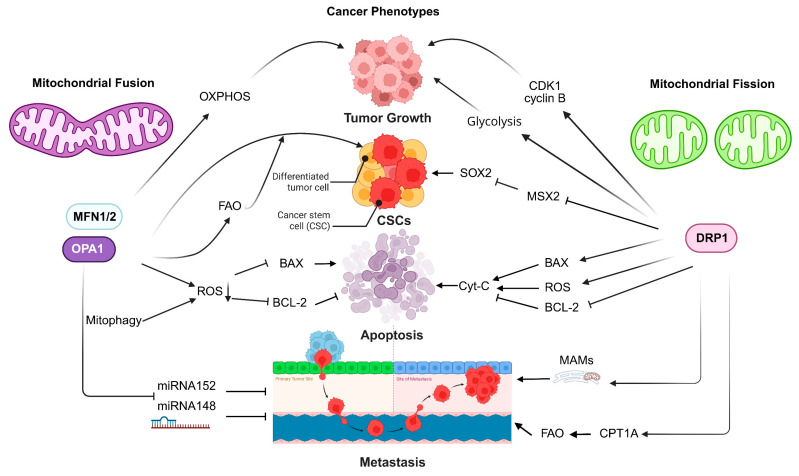
Mitochondrial dynamics in the regulation of cancer cell phenotypes. Mitochondrial fusion (**left**) and fission (**right**) contribute to the regulation of multiple cancer-associated phenotypes, including tumor growth, cancer stemness, apoptosis, and anoikis resistance during metastasis. Both mitochondrial fusion and fission are associated with promoting tumor growth, cancer stemness, and anoikis resistance during metastasis, as indicated by the activation arrows. In contrast, inhibition of cellular apoptosis is represented by the inhibitory arrows. The arrows (→) indicate activation or positive regulatory effects, while inhibitory arrows (⊣) indicate inhibition or negative regulatory effects. Figures were created using BioRender (BioRender.com).

Collectively, mitochondrial dynamics are regulated by multiple cellular signaling pathways, thereby modulating metabolic reprogramming and transcription, leading to cancer cell proliferation and survival (Figure 4).

### 5.2. Stemness and Cellular Plasticity

Cancer cells often acquire resistance to anticancer therapies, and mitochondrial dynamics play critical roles in drug resistance, stemness, and cellular plasticity by promoting metabolic adaptation and survival signaling. In one study, the mitochondrial Complement component 1 Q subcomponent-binding protein (C1QBP) was shown to promote cisplatin resistance by upregulating OPA1, thereby increasing mitochondrial fusion and cancer stemness [115]. In some cases, drug treatment itself can enrich cancer stem cell (CSC) populations. For example, cisplatin treatment has been reported to increase CSC levels. At the mechanistic level, cisplatin induces clusterin expression, which activates AKT-mediated DRP1 phosphorylation, leading to mitochondrial fission. This process promotes mitophagy-mediated degradation of Msh homeobox 2 (MSX2), a SOX2-driven stemness suppressor, thereby maintaining cancer stemness [116]. Cisplatin treatment can also induce the emergence of mesenchymal stem-like cells, which exhibit increased FAO as an adaptive mechanism. Functionally, natriuretic peptide receptor A (NPRA), which is upregulated in these cells, stabilizes MFN2, thereby enhancing FAO and stemness [84]. In addition to FAO, lipogenesis also plays an essential role in maintaining cancer stemness by regulating mitochondrial dynamics through mitochondrial dynamics-related proteins [117]. Metastatic breast cancer cells display distinct metabolic phenotypes that support stemness and survival. A recent study demonstrated that increased lipid accumulation enhances FAO and mitochondrial fission. Studies further demonstrated that elevated DDX3 expression promotes FAO and DRP1-mediated mitochondrial fission, thereby enhancing CSC properties [86]. Nutrient deprivation can also promote cancer stemness by modulating mitochondrial dynamics. For instance, glutamine deprivation has been shown to induce CSC properties and chemoresistance through DRP1-mediated mitochondrial fragmentation [67]. In another study, OSCC stem cells exhibited increased DRP1 expression, leading to enhanced mitochondrial fragmentation and reduced fusion. Suppression of DRP1 led to mitochondrial elongation and promoted glutaminolysis by converting α-ketoglutarate to glutamate. This metabolic shift induced demethylation of histone H3K27me3, ultimately reducing CSC properties [75]. Mitochondrial fusion proteins have also been implicated in stemness regulation. For example, MFN1 has been shown to promote CSC characteristics in ovarian cancer cells. Ovarian cancer stem cells (OCSC) exhibit increased MFN1 expression, which enhances OXPHOS and supports stemness [118]. Recent evidence further indicates that acetylated Krueppel-Like Factor 5 (KLF5) suppresses Lactamase Beta (LACTB) expression, thereby promoting colorectal cancer (CRC) stemness and inhibiting differentiation by modulating mitochondrial dynamics via the OMA1/OPA1 axis [119]. Overall, CSCs appear to upregulate mitochondrial dynamics-related proteins as an adaptive mechanism to sustain stemness, metabolic flexibility, and therapy resistance across various cancer types (Figure 4) [85,120].

Drug treatment and nutrient deprivation can reshape mitochondrial dynamics, helping cancer cells maintain stemness and resist therapy. In these contexts, cisplatin, glutamine deprivation, and related stressors often promote DRP1-dependent mitochondrial fission, which supports mitophagy, metabolic adaptation, and survival of cancer stem-like cells. By contrast, some resistance states instead favor mitochondrial fusion through proteins such as OPA1 or MFN1/MFN2, which can enhance oxidative phosphorylation to sustain stemness in specific cancers.

### 5.3. EMT, Invasion, and Metastasis

Metastasis is a multistep process involving local invasion and epithelial–mesenchymal transition (EMT), intravasation into blood/lymphatic vessels, survival in circulation as circulating tumor cells (CTCs), extravasation into distant organs, and colonization to form secondary tumors. During circulation, cancer cells experience detachment-induced stress known as anoikis. To survive under these conditions, cancer cells develop adaptive mechanisms that confer resistance to anoikis. Mitochondrial dynamics play an important role in regulating metastasis at these different stages. For example, during anoikis resistance, DRP1 interacts with the Binding immunoglobulin Protein (BIP), leading to increased formation of mitochondria-associated endoplasmic reticulum membranes (MAMs), which help maintain mitochondrial dynamics. Mechanistically, AMPK regulates the activation of the MFF-DRP1 axis, promoting DRP1 localization to MAMs [121]. In ovarian cancer, metastasis has been linked to proinflammatory cytokines such as interleukin-6 (IL-6), which promote DRP1-mediated mitochondrial fission. Importantly, IL-6 activates ERK1/2 signaling, which, in turn, activates DRP1 and contributes to metastatic progression [122]. Mitochondria-localized proteins also contribute to metastatic regulation. For instance, mitochondrial-localized isoforms of angiotensin II AT2 receptor-interacting proteins (ATIPs) have been shown to promote metastasis by regulating mitochondrial dynamics [123]. In addition, post-translational modifications such as SUMOylation have been reported to regulate mitochondrial dynamics and cancer cell metastasis [124]. As noted earlier, metastatic cancer cells often exhibit distinct metabolic phenotypes that support migration and invasion. FAO is frequently elevated in metastatic cells and is regulated, in part, by mitochondrial dynamics. Mechanistically, interferon regulatory factor 2 binding protein 2 (IRF2BP2) is upregulated in metastatic cells and promotes DRP1 activation. Activated DRP1, in turn, enhances Carnitine Palmitoyltransferase 1A (CPT1A) activity, which is required for FAO, thereby promoting metastasis [125]. Similarly, brain-metastasized breast cancer cells exhibit increased DRP1-mediated FAO [126]. Exosomes also play an important role in cancer cell migration. For example, exosomal 4EBP1 promotes TGF-β expression, which is essential for cell migration, while inhibition of tumor suppressor PTEN [111]. Oncogenic proteins such as the Particularly Interesting New Cysteine-Histidine-Rich protein 1 (PINCH1), a focal adhesion-associated protein, also contribute to cell migration. PINCH1 has been shown to regulate DRP1 activation, promoting mitochondrial fission and enhancing cell migration in head and neck squamous cell carcinoma (HNSC) [127]. In esophageal squamous cell carcinoma (ESCC), DRP1 promotes EMT through the PGC1-α-Nrf1/2 signaling axis, further supporting the role of mitochondrial dynamics in EMT regulation [128]. Mitochondrial fusion protein OPA1 has also been implicated in cancer cell migration. In breast cancer, OPA1 promotes migration by downregulating miRNAs of the 148/152 family, which normally act as inhibitors of tumor growth and migration [129]. In conclusion, mitochondrial dynamics regulate metastasis across its multistep cascade, from EMT/invasion, intravasation, anokis-resistant CTC survival, extravasation, and colonization (Figure 4).

### 5.4. Apoptosis and Cell Survival

Mitochondria are central regulators of cell fate, determining whether a cell survives or undergoes apoptosis depending on the cellular context. Mitochondrial dynamics proteins play a critical role in maintaining this balance. In general, mitochondrial fusion supports OXPHOS and reduces ROS production, thereby promoting cell survival. In contrast, under conditions such as metabolic stress or anticancer drug treatment, increased mitochondrial fission is often associated with elevated ROS production and induction of apoptosis. For example, mitochondrial proteins such as B-cell lymphoma 2 (Bcl-2)-associated X protein (BAX) directly interact with DRP1, leading to their co-localization on mitochondria. This interaction promotes cytochrome c release and initiates apoptosis in cancer cells under drug treatment conditions [130] (Figure 4). In another study, tumor necrosis factor-α-induced protein 8-like-3 (TIPE3), a regulator of the balance between cell survival and cell death, was shown to bind Phosphoglycerate Mutase Family Member 5 (PGAM5). PGAM5 subsequently interacts with BAX and components of the ETC, while reducing DRP1-Serine 637 phosphorylation. This results in increased mitochondrial fission and membrane permeabilization, cytochrome c release, and induction of apoptosis [84]. Peroxiredoxin 1 (PRDX1), an antioxidant enzyme that is frequently upregulated in several cancers, including hepatocellular carcinoma, has been implicated in regulating cell survival and apoptosis. In a study, PRDX1 overexpression promoted HCC cell proliferation, whereas PRDX1 knockdown induced apoptosis by activating BAX, caspase-3, caspase-9, and PARP-1. Furthermore, PRDX1 depletion reduced BCL-2 expression and induced mitochondrial fragmentation mediated by DRP1, FIS1, and Dynamin 2 (DYN2), highlighting a role for PRDX1 in mitochondrial dynamics and apoptotic regulation [131]. The detailed mechanism by which PRDX1 promotes BCL2 expression and inhibits BAX activation needs to be further investigated in the future. Additional evidence shows that proteins such as Reticulon 4 (RTN4) and Cytoskeleton-Linking Membrane Protein of 63 kDa (CLIMP-63) regulate the recruitment of BAX to the endoplasmic reticulum and mitochondrial membranes, facilitating cytochrome c release and apoptosis through mitochondrial fission [132]. Mitophagy also contributes to apoptosis regulation. PTEN-induced kinase 1 (PINK1)-mediated mitophagy can suppress apoptosis by reducing ROS levels. Conversely, PINK1 depletion or inhibition leads to Translocase of Outer Mitochondrial Membrane 20 (TOM20) oligomerization and recruitment of BAX to the mitochondrial membrane, promoting cytochrome c release and apoptosis [133].

Mitochondrial dynamics have also been implicated in modulating tumor suppressor pathways, including p53/Drp1-mediated mitochondrial fission, to induce apoptosis [134]. The AKT signaling pathway, a key regulator of cell survival, has been shown to inhibit apoptosis. Recent evidence indicates that mitochondrial dynamics proteins, such as DRP1, are regulated by oncogenic AKT signaling. One study shows that inhibition of AKT-mediated phosphorylation of Forkhead box O3a (FOXO3a) by Regorafenib (REGO), a synthetic oral multi-kinase inhibitor with potent antitumor activity, increases FOXO3a nuclear localization, promotes BCL-2-interacting mediator of cell death (BIM) expression, and activates Bax to recruit Drp1 to mitochondria, leading to mitochondrial fission and apoptosis [135] (Figure 4). Mitochondrial dynamics also support cell survival during energy stress. For instance, glioblastoma cells, which are highly dependent on glutamine, respond to glutamine deprivation by upregulating cyclophilin B (CypB), an adaptor protein for CD147. Interaction between CypB and CD147 activates AKT signaling and induces HIF1α and DRP1 activation, leading to increased expression of Glucose Transporter Type 1 (GLUT1), mitochondrial fission, and enhanced cell survival [136].

The anti-apoptotic protein BCL-2 plays a key role in inhibiting apoptosis. Proteins such as BCL2-interacting protein 3 (BNIP3) can suppress BCL-2, leading to cytochrome c release. This is accompanied by increased DRP1-mediated ROS production and reduced MFN1 expression, resulting in decreased OXPHOS and enhanced apoptotic signaling [137] (Figure 4). Remodeling of mitochondrial cristae structure, primarily regulated by OPA1, is a critical determinant of apoptotic sensitivity. It has been shown that OPA1 inhibition induces cytochrome c release and restores sensitivity to anti-Bcl-2 therapy [138]. Collectively, mitochondrial dynamics enable cancer cells to finely balance survival and death signaling pathways, thereby contributing to tumor persistence and resistance to therapy.

Beyond apoptosis, mitochondrial dynamics have also been implicated in other forms of programmed cell death, including gasdermin (GSDME)-mediated pyroptosis and ferroptosis in various cancer models. For instance, cisplatin, transported by Cytochrome C Oxidase Assembly Homolog 17 (COX17) into cochlear mitochondria, binds Myosin IIA, upregulating DRP1 phosphorylation at Serine 616 and downregulating fusion proteins Mfn1, Mfn2, and OPA1 to promote mitochondrial fission. This shift toward fission increases mitochondrial ROS release, driving pyroptosis and cisplatin-induced cytotoxicity in cochlear hair cells [91]. Praja Ring Finger Ubiquitin Ligase (PJA1) promotes docetaxel resistance in nasopharyngeal carcinoma by ubiquitinating PGAM5 for degradation, thereby enhancing DRP1 phosphorylation at Serine 637 and reducing mitochondrial ROS production. This alteration suppresses mitochondrial dysfunction and GSDME-mediated pyroptosis, thereby inhibiting the anti-tumor immune response [139]. Ruxolitinib (Ruxo) inhibits Janus Kinase 1/2 (JAK1/2)-STAT3 signaling in anaplastic thyroid carcinoma (ATC) cells, repressing DRP1 transactivation and causing mitochondrial fission deficiency. This disruption of mitochondrial dynamics blocks GSDME-mediated pyroptosis [140]. Rosmarinic acid (RA) disrupts mitochondrial dynamics in TNBC cells by significantly upregulating DRP1, thereby promoting mitochondrial fission and leading to mitochondrial dysfunction. This fission, coupled with decreased mitochondrial membrane potential, contributes to RA-induced apoptosis and the inhibition of Triple-Negative Breast Cancer (TNBC) cell proliferation [141].

In summary, mitochondrial dynamics have emerged as central regulators of cancer cell phenotypes, including proliferation, stemness, metastasis, and apoptosis. Continued investigation into the molecular mechanisms linking mitochondrial remodeling with oncogenic signaling will be essential to establish their clinical relevance and potential as effective therapeutic targets.

### 5.5. Cellular Senescence and Aging

While acute mitochondrial remodeling enables cancer cells to rapidly adapt to metabolic stress and fluctuating nutrient conditions, chronic disruption of mitochondrial quality control may contribute to cellular senescence and aging-associated phenotypes [142]. Persistent mitochondrial dysfunction, impaired mitophagy, and excessive ROS accumulation can promote senescence-associated secretory phenotypes (SASP), genomic instability, and altered inflammatory signaling [143]. Interestingly, cancer cells may exploit partial senescence-like states to enhance survival and therapy resistance while avoiding irreversible growth arrest. Therefore, mitochondrial dynamics appear to play dual roles in both adaptive metabolic plasticity and long-term degenerative cellular responses. Harmones regulate the mitochondrial dynamics. Dihydrotestosterone (DHT) induces the ROS production by balancing between the activated Phosphor-Drp1 (Ser616) and the inhibitory Phosphor-Drp1 (Ser637) form of DRP1 [144].

Beyond promoting metabolic adaptation and tumor progression, disruption of mitochondrial quality control pathways may also contribute to cellular senescence and aging-associated phenotypes. Chronic alterations in mitochondrial fusion–fission balance, defective mitophagy, and persistent mitochondrial dysfunction can result in excessive reactive oxygen species (ROS) accumulation, mitochondrial DNA damage, impaired ATP production, and activation of stress-responsive signaling pathways [145]. Sustained oxidative stress has been linked to the induction of senescence-associated secretory phenotypes (SASP), chronic inflammation, and irreversible cell cycle arrest, all of which can promote a pro-tumorigenic microenvironment [146]. Furthermore, aging-associated mitochondrial dysfunction may enhance genomic instability, metabolic imbalance, and chronic inflammatory signaling, thereby contributing to cancer initiation and progression. Emerging evidence suggests that cancer cells can exploit senescence-like metabolic states to maintain survival and adapt to prolonged metabolic stress conditions. These observations highlight that mitochondrial remodeling serves as an important link between aging, cellular senescence, and cancer progression.

## 6. Mitochondrial Dynamics in Therapy Resistance

Dysregulation of mitochondrial dynamics, particularly the balance between mitochondrial fusion and fission, plays a critical role in cancer progression and therapeutic resistance. Alterations in these processes disrupt cellular energy homeostasis and promote metabolic reprogramming, including enhanced OXPHOS, glycolysis, and other metabolic pathways. These metabolic adaptations enable tumor cells to suppress apoptosis, maintain survival under stress conditions, and facilitate metastatic progression. Increasing evidence also indicates that mitochondrial dynamics influence the tumor immune microenvironment and contribute to resistance to both conventional chemotherapy and emerging immunotherapies [147].

### 6.1. Fusion-Mediated Therapy Resistance

Accumulating evidence suggests that enhanced mitochondrial fusion can promote tumor cell survival and contribute to therapy resistance in several cancer types. Mitochondrial fusion, driven by MFN1, MFN2, and OPA1, promotes therapy resistance across multiple cancers by preserving mitochondrial integrity, inhibiting cytochrome c release and apoptosis, and reducing ROS/lipid peroxidation. For example, in tamoxifen-resistant breast cancer cells, MFN1 expression has been shown to increase. MFN1 interacts with the cristae organizer OPA1, preventing the release of cytochrome c from mitochondria and thereby inhibiting apoptosis. In addition, MFN1 can interact with the pro-apoptotic protein BCL2 Antagonist/Killer 1 (BAK) and inhibit its oligomerization, further blocking apoptotic signaling and promoting resistance to tamoxifen [148]. Inflammatory signaling pathways may also regulate mitochondrial fusion during therapy resistance. In AML, the pro-inflammatory cytokine IL-6 has been shown to increase MFN1 expression, thereby enhancing mitochondrial fusion and promoting chemoresistance [61]. Similarly, treatment with cisplatin in ovarian cancer has been reported to increase the expression levels of VDAC1 and MFN1. These observations suggest that mitochondrial fusion may be associated with cisplatin resistance, although the precise molecular mechanisms linking VDAC1-mediated mitochondrial regulation and chemoresistance require further investigation [149]. OPA1 has also been implicated in cisplatin resistance in non-small cell lung cancer (NSCLC). Mechanistically, the mitochondrial protein C1QBP1 upregulates OPA1 expression in resistant cells, promoting mitochondrial fusion and thereby contributing to cisplatin resistance [115]. Mitochondrial fusion has also been linked to therapy-induced cellular senescence. Senescence-associated secretory phenotype (SASP) can alter the tumor microenvironment and influence drug responses. Senescent cells were shown to exhibit elevated expressions of MFN1 and MFN2, indicating enhanced mitochondrial fusion. In terms of mechanism, these proteins promote the secretion of immunomodulatory factors, such as galectin-9, into the extracellular environment, suggesting that mitochondrial fusion may influence immune cell infiltration and contribute to therapy resistance [150].

However, reduced mitochondrial fusion could also induce therapy resistance. Recent studies have shown that RANBP2-type and C3HC4-type zinc finger-containing 1 (RBCK1), an E3 ubiquitin ligase, is upregulated in ferroptosis-resistant cells and promotes the ubiquitination and proteasomal degradation of mitofusin 2 (MFN2) in Pancreatic Ductal Adenocarcinoma (PDAC) [151]. Since ferroptosis resistance represents a major obstacle in cancer therapy, loss of MFN2 reduces mitochondrial reactive oxygen species (ROS) production and lipid peroxidation, thereby inhibiting ferroptosis [151], suggesting that reduced mitochondrial fusion confers ferroptosis resistance. In another study, Actin gamma 1 (ACTG1) encodes gamma-actin, is overexpressed in various cancers, suppresses fusion by binding to MFN1, and induces cisplatin resistance [152]. Another E3 ligase, MARCH5, downregulates MFN1 via a ubiquitination-mediated mechanism, leading to mitochondrial dynamics imbalance and venetoclax resistance in multiple myeloma (MM) cells [153]. Superoxide dismutase 2 (SOD2), a mitochondrial antioxidant enzyme that scavenges ROS, has been shown to confer resistance to anlotinib in OSCC. The elevated SOD2 reduces ROS levels and suppresses MFN2 expression, thereby protecting mitochondria from anlotinib-induced damage and promoting anlotinib resistance [154]. Enhanced fusion serves as a convergent resistance node across apoptosis, ferroptosis, senescence, and ROS pathways. However, reduced mitochondrial fusion could also confer therapy resistance by targeting the degradation or suppression of fusion proteins (MFN1/MFN2), leading to reduced ROS and lipid peroxidation. Therefore, mitochondrial fusion thus represents a bidirectional resistance hub in cancer therapy.

### 6.2. Fission-Mediated Therapy Resistance

Similar to mitochondrial fusion, excessive mitochondrial fission has also been implicated in therapy resistance; however, it often promotes tumor progression through distinct mechanisms, including metabolic plasticity, cancer stemness, and adaptation to therapeutic stress. Mitochondrial fission is largely mediated by DRP1, which is recruited to mitochondria through adaptor proteins such as FIS1 and MFF. Oncogenic mutations can influence mitochondrial fission and contribute to therapy resistance. Mutations such as NRAS^Q61R^ and BRAF^V600E^, which are common in melanoma and other cancers, have been associated with altered mitochondrial dynamics. Treatment with the B-Raf Proto-Oncogene, Serine/Threonine Kinase (BRAF) inhibitor vemurafenib has been reported to increase the expression of the fission protein DRP1 while suppressing the fusion proteins MFN1 and MFN2, indicating that mitochondrial fission may be enhanced in vemurafenib-resistant tumor cells [155]. Transcriptional regulators have also been implicated in modulating mitochondrial fission during therapy resistance. In HCC, increased Zinc Finger E-Box Binding Homeobox 1 (ZEB1) expression promotes sorafenib resistance by upregulating DRP1 [156]. Similarly, in cholangiocarcinoma (CCA), resistance to the fibroblast growth factor receptor (FGFR) inhibitor pemigatinib has been linked to increased DRP1 expression. At the molecular level, the kinase Rho Associated Coiled-Coil Containing Protein Kinase 2 (ROCK2) inhibits Ubiquitin-52 Amino Acid Fusion Protein (UBA52) mediated ubiquitin-mediated degradation of DRP1, leading to DRP1 stabilization and enhanced mitochondrial fission in pemigatinib-resistant cells [157]. Altered mitochondrial fission is also observed in castration-resistant prostate cancer (CRPC), an aggressive stage of prostate cancer characterized by therapeutic failure. Darolutamide-resistant cells exhibit increased DRP1 expression and reduced MFN1 levels, suggesting a shift toward enhanced mitochondrial fission [158]. In addition, androgen receptor (AR) inhibitors have been shown to suppress DRP1 phosphorylation and glycolysis; however, resistant cells restore MYC-mediated glycolytic activity along with DRP1 phosphorylation, suggesting that mitochondrial fission contributes to metabolic adaptation during resistance [159]. Mitochondrial fission also plays an important role in cancer stem cells, which are known to exhibit strong resistance to conventional therapies. Studies in osteosarcoma stem cells demonstrated increased DRP1 expression, indicating that enhanced mitochondrial fission may support stem-like properties and drug resistance [160]. The transcription factor Yin Yang 2 (YY2) has been reported to act as a negative regulator of mitochondrial fission by suppressing DRP1 expression, further highlighting the importance of DRP1-dependent pathways in cancer progression and resistance [161]. In addition to regulating metabolism and survival, mitochondrial fission can influence programmed cell death pathways. For example, the E3 ubiquitin ligase PJA1 promotes the degradation of PGAM5, a regulator of DRP1, thereby increasing DRP1 phosphorylation. This process suppresses ROS-mediated pyroptosis and contributes to resistance to docetaxel treatment [139].

Therefore, excessive mitochondrial fission, driven by DRP1 and its regulators (FIS1, MFF, ROCK2, PJA1), is closely linked to therapy resistance across multiple cancers. Oncogenic signals (NRAS^Q61R^, BRAF^V600E^, ZEB1, MYC) and kinase pathways (ROCK2) enhance DRP1 stability or expression, shifting the balance toward fission and away from fusion, thereby supporting metabolic plasticity, cancer stemness, and evasion of ROS-dependent death, such as pyroptosis. In melanoma, HCC, CCA, CRPC, and osteosarcoma, increased DRP1 and reduced MFN1/MFN2 coincide with resistance to targeted agents and chemotherapy, underscoring DRP1-dependent fission as a convergent mechanism that promotes aggressive tumor behavior and treatment failure.

### 6.3. Mitophagy and Metabolic Adaptation in Therapy Resistance

Mitophagy, the selective autophagic removal of damaged mitochondria, represents another critical component of mitochondrial quality control that can influence cancer progression and therapeutic responses. Similar to other aspects of mitochondrial dynamics, mitophagy can function as a double-edged sword in cancer. Under metabolic stress conditions, mitophagy can promote tumor survival by maintaining mitochondrial quality and ensuring the availability of metabolic substrates required for cellular adaptation. Conversely, during early stages of tumor development, mitophagy may suppress tumorigenesis by removing dysfunctional mitochondria and limiting excessive ROS production [162]. Several studies have demonstrated that dysregulation of mitophagy contributes to therapy resistance. Therapy-induced cellular senescence has also been linked to alterations in mitochondrial dynamics and mitophagy. Treatment with 5-Fluorouracil (5-FU) can induce senescence in tumor cells, which contributes to long-term survival and relapse. Senescent cells exhibit increased expression of mitochondrial dynamics proteins, including MFN1, MFN2, FIS1, and DRP1-associated adaptor proteins, suggesting that mitochondrial remodeling may contribute to senescence-associated drug resistance [145]. Metabolic stress conditions can further influence mitochondrial dynamics and mitophagy. For example, glutamine deprivation has been shown to promote glycolytic metabolism and mitochondrial fission by phosphorylating DRP1, highlighting a link between metabolic reprogramming and mitochondrial remodeling in therapy resistance [67]. Loss of tumor suppressor proteins can also enhance mitophagy-mediated therapy resistance. Glutathione S-transferase kappa 1 (GSTK1), a mitochondrial and peroxisomal enzyme, is significantly downregulated in HCC. Loss of GSTK1 promotes mitochondrial fission and mitophagy by facilitating the interaction between PGAM5 and DRP1, thereby enhancing DRP1-mediated mitochondrial fission. Conversely, GSTK1 acts as a tumor suppressor by inhibiting mitochondrial fission by competing with DRP1 for binding to PGAM5, thereby maintaining mitochondrial quality control and inhibiting aberrant mitophagy. Dysregulation of this pathway may contribute to enhanced tumor progression and potential chemotherapy resistance in HCC [158]. Similarly, the tumor suppressor PRKAB2 is markedly reduced in renal cell carcinoma (RCC). Loss of 5′-AMP-Activated Protein Kinase, Beta-2 Subunit (PRKAB2) promotes mitophagy and contributes to resistance to tyrosine kinase inhibitors such as sunitinib. Mechanistically, PRKAB2 forms a complex with Leucine Rich Pentatricopeptide Repeat Containing (LRPPRC) and PRKN to suppress mitophagy and activate AMPK, which in turn inhibits cardiolipin biosynthesis, a lipid required for mitophagy initiation [163]. Emerging evidence further suggests that mitochondrial dynamics and mitophagy influence immune cell function within the tumor microenvironment. Excessive mitochondrial fission driven by the PGAM5-DRP1 signaling axis can induce mitochondrial dysfunction and promote T-cell exhaustion, thereby limiting the efficacy of cancer immunotherapy. Conversely, the E3 ubiquitin ligase Kelch Like Family Member 6 (KLHL6) helps maintain mitochondrial quality control by restraining DRP1-mediated fission and preventing terminal T-cell exhaustion, ultimately enhancing anti-tumor immune responses [164].

Mitophagy drives therapy resistance by reshaping mitochondrial quality control, dynamics, and immune crosstalk. In multiple cancers, dysregulated mitophagy often promotes mitochondrial fragmentation by disrupting tumor suppressors, such as GSTK1 or PRKAB2, thereby enhancing clearance of damaged organelles and supporting metabolic adaptation. Additionally, senescence and stress-induced shifts toward fission and mitophagy reinforce drug-resistant states. In the tumor microenvironment, excessive PGAM5-DRP1-driven fission impairs T cell function. Therefore, mitophagy modulates therapy resistance not only in cancer cells but also in the tumor microenvironment.

## 7. Therapeutic Targeting of Mitochondrial Dynamics

Targeting mitochondrial dynamics has emerged as a promising therapeutic strategy in cancer, as mitochondrial fission, fusion, and mitophagy are critical determinants of tumor cell survival, metabolism, and treatment response. Ceritinib, a tyrosine kinase inhibitor, has been reported to promote mitochondrial fission and ROS production by activating DRP1 and cleaving OPA1, without affecting MFN1 and MFN2 expression levels. Ceritinib activates the mitochondrial calcium uniporter (MCU)/calpain signaling pathway, leading to OPA1 cleavage and DRP1 activation. This process ultimately suppresses thyroid cancer cell growth [165]. Piceatannol (PCT), a hydroxystilbene compound with anti-colitic properties, has also been shown to modulate mitochondrial dynamics. In chemotherapy-induced senescent CRC cells, PCT promotes mitochondrial fission by upregulating the MFF, thereby enhancing cell death [145].

OPA1 is frequently overexpressed in several cancers, including breast cancer (Figure 3), where it contributes to tumor progression and therapeutic resistance. Inhibition of OPA1 has therefore emerged as a potential anti-cancer strategy. Small-molecule inhibitors such as MYLS22 and Opitor-0 have demonstrated promising effects in breast cancer by suppressing cancer cell proliferation and migration [104]. Olaparib, an oral inhibitor of poly(ADP-ribose) polymerase (PARP) widely used in ovarian, breast, prostate, and pancreatic cancers, has also been linked to mitochondrial dynamics. Studies have shown that olaparib promotes CDK5-mediated phosphorylation of DRP1 at Serine 616, resulting in enhanced mitochondrial fission and apoptosis via caspase activation. These findings suggest that promoting mitochondrial fission may contribute to its therapeutic efficacy [166].

Mitophagy also plays a significant role in cancer progression. PINK1-mediated mitophagy is essential for the removal of damaged mitochondria and the regulation of ROS levels, thereby influencing both cell survival and death pathways. In many tumors, elevated or dysregulated mitophagy serves an oncogenic function by promoting cancer cell fitness under stress conditions. By selectively eliminating dysfunctional mitochondria, mitophagy reduces excessive ROS and prevents activation of mitochondrial-dependent cell death, allowing cancer cells to survive metabolic stress, hypoxia, and genotoxic insults such as chemotherapy or radiation [167]. Inhibition of PINK signaling has been explored as a therapeutic strategy. For example, the PINK inhibitor Quizartinib (AC220) induces ROS production, promotes TOMM20 oligomerization, and triggers BAX activation. These events lead to cytochrome c release and caspase-3 activation, followed by caspase-3-mediated cleavage of Gasodermin, ultimately inducing pyroptotic cell death [168]. Donafenib, a multikinase inhibitor approved for HCC, has also been shown to influence mitochondrial dynamics. On a mechanistic basis, Donafenib suppresses glutathione peroxidase 4 (GPx4) expression and increases ROS production in HCC cells. In addition, it induces DRP1 expression without significantly affecting MFN1 or MFN2 levels, indicating that it promotes mitochondrial fission-mediated cell death [169].

Certain therapeutic agents may exert context-dependent effects on mitochondrial dynamics. For instance, Quercetin has demonstrated anti-cancer activity in several malignancies, including HCC. In HCC cells, Quercetin treatment promotes mitochondrial fusion by upregulating MFN1 and MFN2 while reducing DRP1 and FIS1-mediated fission. Additionally, it enhances PINK-1 and PRKN-dependent mitophagy. Hence, quercetin-induced mitochondrial fusion and PINK1/PRKN-dependent mitophagy may negatively influence its anticancer effects in HCC. These observations suggest that combining Quercetin with mitophagy inhibitors may represent a promising therapeutic approach [170]. In some contexts, mitochondrial fusion itself can help regulate ROS production and suppress excessive mitophagy, highlighting the complexity of mitochondrial dynamics in cancer [171]. Protodioscin (PD), a steroidal saponin, has also been reported to induce apoptosis in HCC models. Mechanistically, PD enhances MFN1 expression and promotes its interaction with the pro-apoptotic protein BAK at the endoplasmic reticulum (ER), facilitating calcium influx into mitochondria and triggering apoptotic signaling [172]. Post-translational regulation of mitochondrial fusion proteins is another emerging therapeutic target. MFN1 ubiquitination is promoted by RAN-binding protein 9 (RANBP9), a core component of the C-terminal to LisH (CTLH) E3 ligase complex, with the assistance of FAM111 Trypsin Like Peptidase B (FAM111B). Targeting this pathway with glypican-3 (GPC3)-targeted lipid nanoparticles for the efficient delivery of siFAM111B has shown promising effects, promoting mitochondrial fusion and inhibiting mitophagy [173].

Similarly, E3 ubiquitin ligases such as MARCH5 can downregulate MFN1 through ubiquitination-mediated degradation, thereby disrupting mitochondrial dynamics [151]. Consequently, pharmacological activation of MFN1, for example, using leflunomide, may represent a potential therapeutic strategy in certain cancers by targeting the MARCH5-MFN2 mitochondrial axis, thereby promoting mitochondrial fusion and inhibiting mitophagy [62,153]. In addition to MFN1 and DRP1, other mitochondrial dynamics regulators such as OPA1 and MFN2 also contribute to cancer progression and therapeutic resistance. TMQ0153, a tetrahydrobenzimidazole compound, has shown promising activity in AML by reducing the expression of MFN2 and OPA1, suggesting that simultaneous targeting of these fusion proteins may provide therapeutic benefit [64]. Mitochondrial Division Inhibitor-1 (Mdivi-1) is another compound widely used to modulate mitochondrial dynamics in various diseases, including cancer. In CRC models, Mdivi-1 has been shown to decrease DRP1 protein levels while increasing MFN2 expression, thereby suppressing tumor invasion and metastasis [174]. As discussed earlier, mitophagy plays a dual role in cancer, functioning as a “double-edged sword.” Depending on the cellular context, mitophagy can either promote tumor cell survival or contribute to tumor suppression. Therefore, both mitophagy inducers and inhibitors may have therapeutic potential, although their application requires careful consideration of tumor type and disease stage.

**Figure 5:**
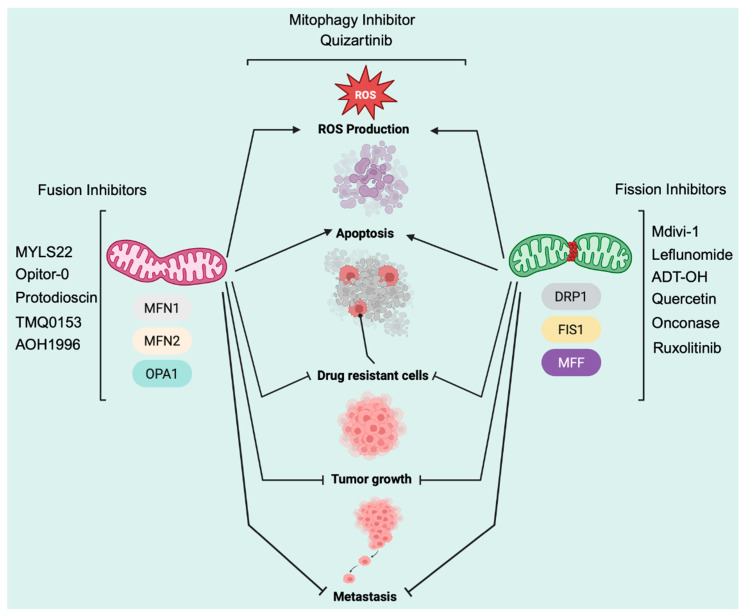
Therapeutic targeting of mitochondrial dynamics in cancer. Recent therapeutic strategies target mitochondrial dynamics processes, including fusion, fission, and mitophagy, across various cancers. Inhibitors of mitochondrial fission, fusion, or mitophagy can increase ROS production, induce apoptosis, overcome drug resistance, suppress tumor growth, and metastasis. Figures were created using BioRender (BioRender.com).

For example, Tubeimoside I has been reported to induce mitophagy via the PINK1/PRKN/MFN2 signaling pathway, thereby inhibiting AML cell proliferation [175]. Another emerging therapeutic approach involves targeting protein interactions that regulate mitochondrial fusion. AOH1996, a small-molecule inhibitor of PCNA, has been shown to disrupt the interaction between PCNA and the mitochondrial fusion protein OPA1 in AML. As PCNA stabilizes OPA1, AOH1996 disrupts this interaction, allowing the E3 ubiquitin ligase MARCH5 to ubiquitinate and degrade OPA1. This leads to reduced mitochondrial fusion and OXPHOS, ultimately suppressing the growth of leukemia stem cells (LSCs) [65]. In addition to these examples, several other compounds targeting mitochondrial dynamics regulators, including MFN1, MFN2, OPA1, DRP1, and related pathways, have been identified (Figure 5). These agents are summarized in Table 1 and highlight the growing therapeutic potential of modulating mitochondrial dynamics in cancer treatment.

In addition, repurposed mitochondrial-targeting agents such as doxycycline, metformin, and leflunomide have emerged as modulators of mitochondrial function, dynamics, and metabolic plasticity in cancer. Doxycycline, a tetracycline antibiotic, inhibits mitochondrial biogenesis, which is required for cancer stemness, thereby impairing tumor cell proliferation and EMT [191,192]. In glioblastoma cells, doxycycline has been shown to induce mitochondrial dysfunction and oxidative stress, decrease PCNA levels, thereby suppressing tumor growth and enhancing the response to chemotherapy [193]. Similarly, metformin, a widely used antidiabetic drug, inhibits mitochondrial complex I and activates AMPK signaling, leading to metabolic stress and mitochondrial remodeling in cancer cells [194]. Recent studies further indicate that metformin induces adaptive changes in mitochondrial dynamics, including modulation of the fusion–fission balance and mitochondrial biogenesis, and induction of apoptosis through VDAC1 oligomerization [195,196]. Leflunomide, an inhibitor of dihydroorotate dehydrogenase (DHODH), disrupts mitochondrial metabolism and has been associated with modulation of mitochondrial dynamics and suppression of cancer cell proliferation and metastasis [197,198]. In various cancer models, leflunomide has been reported to reduce DRP1 phosphorylation, decrease mitochondrial fragmentation, and suppress tumor growth [197]. Collectively, these findings highlight that clinically approved drugs can converge on mitochondrial function, supporting mitochondrial dynamics and metabolism as actionable vulnerabilities in cancer.

## 8. Conclusions and Future Directions

Emerging evidence suggests that mitochondrial remodeling in cancer is not solely a passive consequence of transformation but may represent an actively regulated adaptive program shaped by both oncogenic signaling and selective pressures within the tumor microenvironment. Oncogenic pathways directly modulate mitochondrial fusion, fission, and mitophagy machinery, whereas hypoxia, nutrient limitation, oxidative stress, and therapeutic stress selectively enrich tumor cell populations with mitochondrial phenotypes that enhance metabolic plasticity, stemness, survival, and metastatic potential. Thus, mitochondrial dynamics likely represent highly plastic and reversible adaptive programs that facilitate tumor evolution and therapeutic resistance.

Despite increased understanding of mitochondrial dynamics in various cancers, many important questions remain unresolved. A deeper mechanistic understanding of how mitochondrial fission, fusion, and mitophagy coordinate with oncogenic signaling pathways will be essential for translating these findings into clinically effective therapeutic strategies. One key challenge in targeting the mitochondrial dynamics is the context-dependent role of mitochondrial dynamics machinery in cancer progression. Mitochondrial fission can promote tumor progression, metastasis, and metabolic adaptation in certain cancers, whereas in others it can trigger mitochondrial dysfunction, oxidative stress, and apoptosis. Similarly, mitochondrial fusion may support metabolic plasticity and tumor survival, and help prevent excessive mitochondrial damage by mitigating ROS accumulation. Therefore, future studies should aim to define cancer type or context-dependent mitochondrial dynamics signatures that determine whether targeting fission or fusion will provide therapeutic benefit.

Another important direction involves deciphering the interplay between mitochondrial dynamics and cancer metabolism. Mitochondrial morphology is closely linked to metabolic rewiring, including OXPHOS, glycolysis, FAO, glutaminolysis, and nucleic acid synthesis. Drug-resistant cancer cells often exhibit profound metabolic plasticity, and mitochondrial dynamics may support this plasticity. Integrative approaches combining targeted and untargeted metabolomics, such as isotope labeling experiments, mitochondrial bioenergetic profiling (OCR and EACR), and live-cell imaging using the mitochondrial tracker dyes, could provide valuable insight into how mitochondrial dynamics contribute to metabolic adaptation and therapy resistance.

Recent evidence also highlights the critical role of mitophagy in sustaining mitochondrial quality control during cancer progression and therapeutic stress. However, mitophagy emerges as a double-edged sword. While it can protect tumor cells by scavenging ROS, mitophagy is under control; excessive mitophagy may lead to bioenergetic collapse and cell death. Future research should therefore focus on identifying molecular signaling pathways that define the threshold between favorable and unfavorable mitophagy, thereby guiding the development of therapeutic strategies that selectively employ mitophagy in cancer cells.

Another encouraging area involves targeting mitochondrial dynamics to overcome therapy resistance. Many chemotherapeutic mediators and targeted therapies induce mitochondrial ROS and mitochondrial fission in cancer cells [64,128,168]. Cancer cells frequently adapt to these stresses through mitophagy to scavenge ROS, promote the expression of certain oncogenes for survival, and activate mitochondrial quality control pathways [199]. Combining conventional anti-cancer therapies with small-molecule modulators of mitochondrial fission, fusion, or mitophagy may therefore represent a powerful strategy to re-sensitize resistant tumors. Rationally designed combination therapies that exploit mitochondrial vulnerabilities may significantly improve treatment outcomes.

Recent advances in structural biology and drug discovery technologies also open new opportunities for targeting mitochondrial dynamics proteins. High-resolution structural studies of proteins such as DRP1, OPA1, MFN1, and MFN2 are beginning to reveal potential druggable interfaces [200,201,202,203]. Structure-guided drug design, together with high-throughput chemical screening, may enable the development of highly selective inhibitors or activators with improved specificity and reduced off-target toxicity. Several drugs regulate mitochondrial dynamics through fusion, fission, or mitophagy (Table 1), and the use of these techniques will lead to the identification of lead compounds for each cancer type; further research is warranted at this stage.

It is worth noting that mitochondrial dynamics should also be investigated within the broader context of the tumor microenvironment and cancer stem cell biology. Increasing evidence suggests that mitochondrial dynamics in immune cells, stromal cells, and CSCs may influence immune evasion, metastasis, and therapeutic response. Understanding these interactions may reveal new opportunities to integrate mitochondrial dynamics-targeted immunotherapy with microenvironment-directed therapies, thereby enabling strategies that both inhibit cancer cell growth and enhance anti-tumor activity. Advanced techniques such as multi-omics, single-cell sequencing, and metabolomics should be employed to accelerate progress in this field. These approaches will allow researchers to examine mitochondrial dynamics with unprecedented resolution and may uncover previously unrecognized heterogeneity in mitochondrial remodeling across tumor cell populations.

It has been observed that the mitochondrial dynamics machinery is highly overexpressed in various cancers [204] (Figure 3), and these machinery proteins can be used as biomarkers after careful investigation. More importantly, mitochondrial morphology itself may serve as a predictive biomarker of therapy resistance, helping identify tumors with distinct mitochondrial fusion, fission, or fragmentation states associated with treatment failure. In addition, biomarkers based on mitochondrial protein expression, metabolic signatures, or mitophagy activity may further improve patient stratification and help identify those most likely to benefit from therapies targeting mitochondrial dynamics.

Future studies should further explore the translational potential of mitochondrial dynamics in cancer stratification and precision medicine. Emerging mitochondrial remodeling signatures, including altered expression and activity of key regulators such as DRP1, MFN1/2, OPA1, and mitophagy-related proteins, may define biologically and functionally distinct tumor subtypes characterized by specific metabolic states, metastatic potential, and therapeutic vulnerabilities. These mitochondrial features may therefore provide a framework for refining patient stratification and guiding personalized therapeutic strategies.

Mitochondrial dynamics hold considerable promise as a source of clinically relevant biomarkers, particularly in cancer, but their most realistic application will be as part of multi-parameter signatures rather than standalone readouts. Alterations in the expression, post-translational modification, or localization of key fission and fusion regulators (such as DRP1, MFN1/2, OPA1, and FIS1), together with quantitative measures of mitochondrial morphology and mitophagy activity, closely mirror shifts between glycolytic and OXPHOS-dependent states, therapy resistance programs, and cancer stemness, suggesting value for prognostic and predictive biomarker development. In practical terms, it is likely that composite panels integrating mitochondrial dynamics genes and proteins with ex vivo functional assays (for example, bioenergetic profiling, mitophagy markers), metabolomic features (such as lactate and TCA intermediates), and microenvironmental context will provide the most robust stratification of tumor subtypes and treatment responses.

In parallel, artificial intelligence (AI) and machine learning approaches are increasingly being recognized as powerful tools for integrating complex multi-dimensional datasets. AI-driven analysis of genomic, transcriptomic, metabolomic, spatial imaging, and single-cell data may facilitate the identification of conserved and context-specific mitochondrial remodeling patterns underlying tumor heterogeneity and therapy response. Such computational frameworks could enhance patient stratification and support the development of personalized therapeutic approaches targeting mitochondrial vulnerabilities in defined cancer subgroups.

Collectively, integrating mitochondrial dynamics with multi-omics profiling, computational and AI-driven modeling, cancer metabolism, drug discovery, and precision oncology holds strong promise to transform mechanistic insights into clinically actionable strategies. This integration may enable more precise cancer diagnosis, improved patient stratification, and effective targeting of mitochondrial vulnerabilities in cancer therapy.

In terms of targeting mitochondrial dynamics, it has been developed so that DRP1 inhibitors such as P110, Drpitor1, Drpitor1a, Dynasore, MIDI, and Mdivi-1 effectively block DRP1 and related dynamin family proteins. These agents show therapeutic promise across diverse pathologies, including Alzheimer’s disease, acute kidney injury, and diabetes [205,206,207,208], yet their anticancer potential remains largely untapped. Future research should prioritize evaluating these inhibitors’ ability to induce apoptosis by modulating mitochondrial dynamics across cancer models, potentially revealing novel vulnerabilities for clinical translation. A comprehensive understanding of the molecular mechanisms governing mitochondrial dynamics in cancer may ultimately pave the way for mitochondria-targeted therapies capable of overcoming tumor progression and therapy resistance.

## Figures and Tables

**Table 1:** Various drugs related to the mitochondrial dynamics in cancer.

Drug	Effect on Mitochondrial Dynamics	Type of Mitochondrial Dynamics	Cancer Model	References
Ceritinib	Increased DRP1 levels, reduced OPA1 levels	Promotes fission	Thyroid Cancer	[165]
Piceatannol (PCT)	Increased MFF levels, downregulated MFN1/2	Promotes fission	CRC	[145]
MYLS22	Inhibits OPA1	Suppress the fusion	Lung and Breast Cancer	[129,176]
MYLS22 and Opitor-0	Inhibits OPA1	Suppress the fusion	Breast Cancer	[104,138]
Olaparib	Promotes CDK5-mediated phosphorylation of DRP1 at Serine 616	Promotes fission	Ovarian Cancer	[166]
Quizartinib (AC220)	Inhibits PINK-mediated mitophagy	Inhibits mitophagy	Neuroblastoma	[168]
Donafenib	Induces the activation of DRP1	Promotes fission	Liver Cancer	[169]
Quercetin	Increases MFN1/2 and PINK1/Parkin expression; decreases DRP1 and FIS1 expression	Promotes fusion and mitophagy	HCC, CRC	[170,177]
Protodioscin	Mfn1–Bak–IP3R complex formation	Promotes fission	HCC	[172]
Glypican-3 (GPC3)-targeted lipid nanoparticles for siFAM111B delivery	Stabilizes MFN1 levels	Promotes fusion	HCC	[173]
Leflunomide	Activates MFN1	Promotes fusion	Multiple Myeloma	[153]
TMQ0153	Decreased OPA1 and MFN2 expression	Promotes fission	AML	[64]
Mdivi.1	Decreased DRP1 and increased MFN2	Promotes fusion	CRC	[174]
Mdivi.1	Decreased DRP1 and Increased MFN2	Promotes fusion	Glioma	[178]
Tubeimoside I	Decreased MFN2 levels	Promotes Fission and mitophagy	AML	[175]
Ellagic Acid (EA)	Decreased MFN2 and total DRP1 levels	Promotes ROS production	Ovarian Cancer	[179]
ADT-OH	Decreased DRP1 and Increased MFN2	Promotes fusion	Breast Cancer	[180]
Paeonol (Pae)	Increased MFN2 expression	Promotes fusion	Primary Cardiomyocytes	[181]
SAHA	PRKN acetylation-mediated mitophagy	Promotes mitophagy	Cervical Cancer	[182]
Bufalin	Affects the translocation of DRP1 and MFN2 between the cytosol and mitochondria.	Promotes fusion	Glioma	[178]
Onconase (ONC)	Reduced PGC1α, DRP1, and FIS1 expression	Promotes fusion	Melanoma	[183]
Bromoxib	Cleaves the OPA1	Promotes fission	Leukemia, Lymphoma, and Glioblastoma	[184]
CTU	Promotes the OMA1 mediated OPA1 cleavage	Promotes fission	Breast Cancer	[185]
STM2457	Inhibits m^6^A modification of OPA1	Promotes fission	CRC	[186]
BTM-3566 BTM-3528	Promotes the OMA1 mediated OPA1 cleavage	Promotes fission	B-cell Lymphoma	[187]
Viriditoxin (VDT)	Cleavage of OPA1	Promotes fission	Leukemia and Lymphoma	[188]
Liensinine	Dephosphorylates DRP1-Ser637	Promotes fission	Lung adenocarcinoma	[189]
Ruxolitinib	Decreased total DRP1 levels	Mitochondrial dysfunction	Thyroid carcinoma	[140]
Nujiangexanthone A	Degrades the fusion proteins MFN1 and MFN2	Promotes mitophagy	Cervical Cancer	[190]
Rosmarinic acid	Activates the DRP1	Promotes fission	Triple Negative Breast Cancer (TNBC)	[141]
Reniformin A	Promotes the association of DRP1 with Bax	Mitochondrial dysfunction	TNBC	[130]

## Data Availability

The data used in this article were obtained from online databases, including TIMER and GEPIA. Hence, data can be retrieved using these databases by following the references mentioned in this article.
